# Blood glucose-related indicators are associated with in-hospital mortality in critically ill patients with acute pancreatitis

**DOI:** 10.1038/s41598-021-94697-1

**Published:** 2021-07-28

**Authors:** Yan Lu, Qiaohong Zhang, Jianjie Lou

**Affiliations:** Clinical Laboratory, DongYang People’s Hospital, 60 West Wuning Road, Dongyang, 322100 Zhejiang China

**Keywords:** Medical research, Risk factors

## Abstract

Acute pancreatitis (AP) results in potentially harmful blood glucose fluctuations, affecting patient prognosis. This study aimed to explore the relationship between blood glucose-related indicators and in-hospital mortality in critically ill patients with AP. We extracted data on AP patients from the Multiparameter Intelligent Monitoring in Intensive Care III database. Initial glucose (Glucose_initial), maximum glucose (Glucose_max), minimum glucose (Glucose_min), mean glucose (Glucose_mean), and glucose variability (glucose standard deviation [Glucose_SD] and glucose coefficient of variation [Glucose_CV]) were selected as blood glucose-related indicators. Logistic regression models and the Lowess smoothing curves were used to display the association between significant blood glucose-related indicators and in-hospital mortality. Survivors and non-survivors showed significant differences in Glucose_max, Glucose_mean, Glucose_SD, and Glucose_CV (*P* < 0.05). Glucose_max, Glucose_mean, Glucose_SD, and Glucose_CV were risk factors for in-hospital mortality in AP patients (OR > 1; *P* < 0.05). According to the Lowess smoothing curve, the overall trends of blood glucose-related indicators showed a non-linear correlation with in-hospital mortality. Glucose_max, Glucose_mean, Glucose_SD, and Glucose_CV were associated with in-hospital mortality in critically ill patients with AP.

## Introduction

Blood glucose regulation is an important part of human endocrine regulation. When the human body suffers from a severe illness or severe injury, it activates the neuroendocrine system and causes the secretion of numerous stress hormones, leading to an increase in blood glucose levels^1^. However, in patients with severe acute pancreatitis (AP), many pancreatic cells are damaged^2,3^, and thus insufficient insulin secretion can cause stressful blood glucose fluctuations^4^.

According to some studies, transient stress hyperglycemia in critically ill patients is considered harmless, indicating that the body has normal immune regulation capabilities^5^; nonetheless, fluctuations in blood glucose are considered to cause irreversible organ damage and affect patient prognosis^6,7^. However, there are no blood glucose-related indicators for the clinical treatment of patients with AP. Therefore, this study aimed to explore the relationship between blood glucose-related indicators and in-hospital mortality in critically ill patients with AP.

## Materials and methods

### Data source

This study was based on the Multiparameter Intelligent Monitoring in Intensive Care III (MIMIC-III, v1.4) database^8^. This is a large, free database open to researchers worldwide, and it contains clinical data of more than 40,000 patients who stayed in the ICU of the Beth Israel Deaconess Medical Center between 2001 and 2012^8^. The data in the MIMIC-III database is anonymous, so there is no need to obtain informed consent from all subjects. The author, *Lu*, completed the Protecting Human Research Participants (record ID: 35953547) online exam and gained access to the MIMIC-III database.

### Study population and data extraction

The personal information and clinical data of all patients were extracted from the MIMIC-III database using the structured query language. All adult patients (≥ 18 years of age) with AP were recruited. Patients were excluded when more than 5% of their key data were missing. When the patient was repeatedly admitted to the ICU for AP, only data regarding the first admission was evaluated. All methods were carried out in accordance with relevant guidelines and regulations.

We extracted data on age, sex, comorbidities (diabetes, hypertension, coronary heart disease, and chronic obstructive pulmonary disease), admission time, survival status, initial severity scores after admission to the ICU (sequential organ failure assessment [SOFA] score^9^, systemic inflammatory response syndrome [SIRS] score^10^, and Oxford Acute Severity of Illness Score [OASIS]^11^), white blood cell count, platelet count, creatinine concentration, and blood glucose level. For the white blood cell count, platelet count, and creatinine concentration, we extracted data for first measurement after admission to the ICU. The outcome of the study was in-hospital mortality, which was defined as death during hospital stay.

We extracted all blood glucose measurement values recorded after admission and included them based on the following criteria: (1) the timing of detection was within 120 h after admission; (2) the first test result after admission was used as the initial value of blood glucose level, and then the value was included once in 23–48 h; and (3) the glucose value was measured at least three times. Patients with blood glucose measurement values that did not meet the above criteria were excluded. The values of six blood glucose-related indicators were included in the study: initial glucose (Glucose_initial), maximum glucose (Glucose_max), minimum glucose (Glucose_min), mean glucose (Glucose_mean), glucose standard deviation (Glucose_SD), and glucose coefficient of variation (Glucose_CV). Accordingly, the Glucose_CV and Glucose_SD constituted glucose variability parameters, with the Glucose_CV being the ratio of the Glucose_SD to the mean.

### Statistical analysis

Data were exported to Stata (version 12.0; Stata Corp, College Station, TX). If the continuous variable entailed normally distributed data, it was expressed as the mean ± SD. In contrast, non-normally distributed data were expressed as medians and interquartile ranges (IQRs). The chi-square test was used to compare categorical variables^12^. We used the t-test^13^ and Wilcoxon rank-sum test^14^ to compare parametric and nonparametric continuous variables, respectively. A *P*-value of < 0.05 was considered statistically significant. A logistic regression model was used to determine the association between statistically significant blood glucose-related indicators and in-hospital mortality; the data were expressed as odds ratios (ORs) with 95% confidence intervals (CIs). For blood glucose-related indicators that were significantly associated with AP-related in-hospital mortality, we used Lowess smoothing curves to determine the overall trend of their correlation with in-hospital mortality.

We used SPSS (version 22.0; IBM SPSS Statistics Inc., 2012, IBM, Chicago, IL) to construct the receiver operating characteristic (ROC) curve. The area under the ROC curve (AUC) was used to evaluate the predictive ability of critically ill patients with AP^15^.

## Results

### Population characteristics

**Figure 1:**
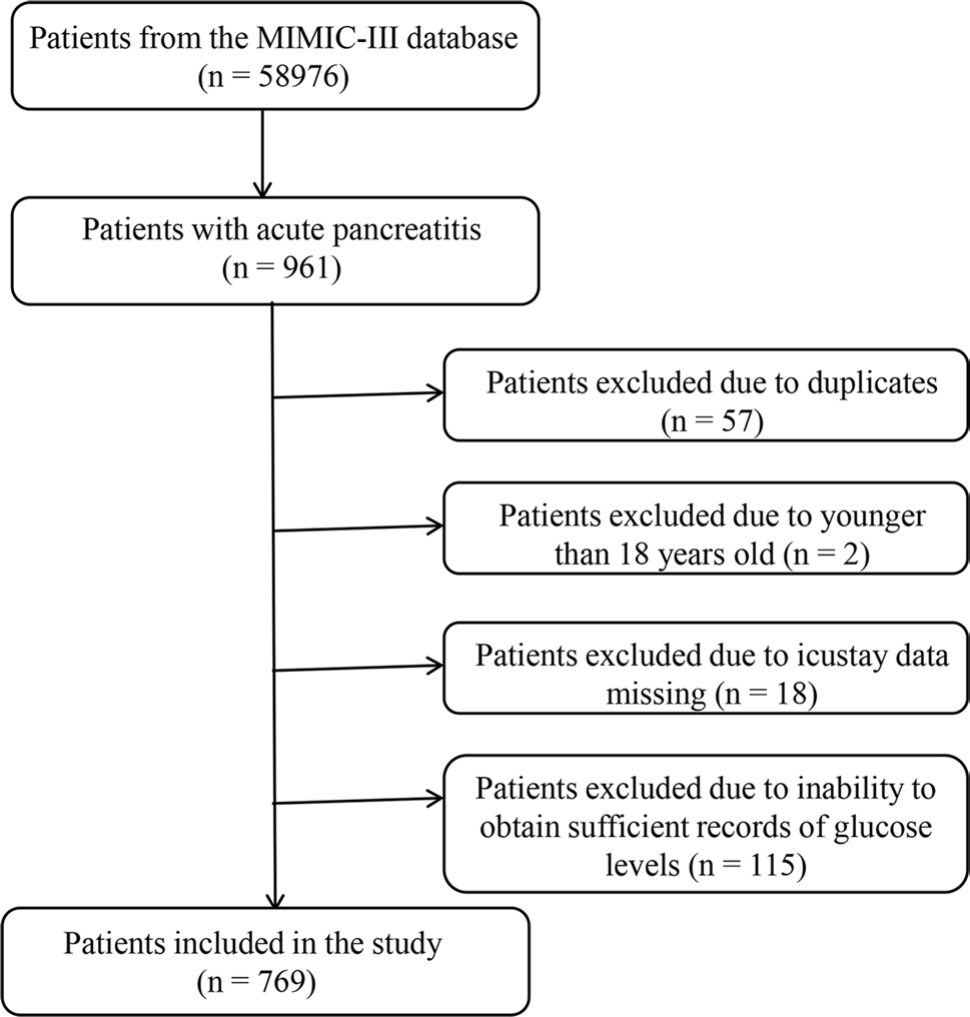
Flow chart of the study selection process.

After reviewing 58,976 admissions in the MIMIC-III database, a total of 769 patients were included in the study (Fig. 1). Table 1 shows the characteristics of the study population. The median age of the study population was 59.43 years, and 55.0% were men. There were 663 survivors and 106 non-survivors. Compared to non-survivors, survivors were younger and had lower severity scores. Although Glucose_max, Glucose_mean, Glucose_CV, and Glucose_SD were related to in-hospital mortality, Glucose_initial and Glucose_min were not.

**Table 1:** Characteristics of patients with acute pancreatitis.

	Total (n = 769)	Non-survivors (n = 106)	Survivors (n = 663)	*P*-value
Age, years (IQR)	59.43 (46.58–72.50)	67.61 (53.16–80.50)	58.18 (45.90–71.02)	< 0.001
Male, n (%)	423 (55.0)	40 (37.7)	306 (46.2)	0.106
**Comorbidities, n (%)**				
Diabetes	220 (28.6)	35 (33.0)	185 (27.9)	0.279
Hypertension	314 (40.8)	41 (38.7)	273 (41.2)	0.627
Coronary heart disease	109 (14.2)	20 (18.9)	89 (13.4)	0.136
COPD	9 (1.2)	0 (0.0)	9 (1.4)	0.228
**Severity, scores (IQR)**				
SOFA	5 (3–7)	8 (5–11)	4 (2–7)	< 0.001
SIRS	3 (3–4)	4 (3–4)	3 (3–4)	0.003
OASIS	32(26–39)	39 (32–46)	31 (25–38)	< 0.001
WBC, 10 ^9^/L (IQR)	12.6 (8.6–17.6)	13.9 (8.0–17.7)	12.4 (8.6–17.6)	0.6628
Platelet, 10 ^9^/L (IQR)	233 (156–348)	172.5 (74–275)	243 (165–360)	< 0.001
Creatinine, mg/dL (IQR)	1.1 (0.8–1.9)	1.25 (0.8–2.1)	1.1 (0.8–1.8)	0.061
Glucose_initial, mg/dL (IQR)	117 (95.5–148)	122 (101–151)	115.5 (95–147)	0.109
Glucose_max, mg/dL (IQR)	149 (124–190)	170.5 (139–213)	145 (122–184)	< 0.001
Glucose_min, mg/dL (IQR)	94 (80–113)	95.5 (80–114)	93 (80–112)	0.249
Glucose_mean, mg/dL (IQR)	120.5 (103.75–144.75)	128.33 (114.8–167.5)	119.2(101.2–142.5)	< 0.001
Glucose_SD, mg/dL (IQR)	23.05 (13.66–39.82)	34.34 (19.31–48.83)	21.93 (13.10–37.54)	< 0.001
Glucose_CV, % (IQR)	19.12 (11.94–30.94)	24.84 (15.17–35.07)	18.55 (11.51–29.92)	< 0.001

*IQR* interquartile range, *COPD* chronic obstructive pulmonary disease, *SOFA* sequential organ failure assessment, *SIRS* systemic inflammatory response syndrome, *OASIS* Oxford acute severity of illness score, *WBC* white blood cell, *SD* standard deviation, *CV* coefficient of variation.

### Predictive abilities of blood glucose-related indicators

**Figure 2:**
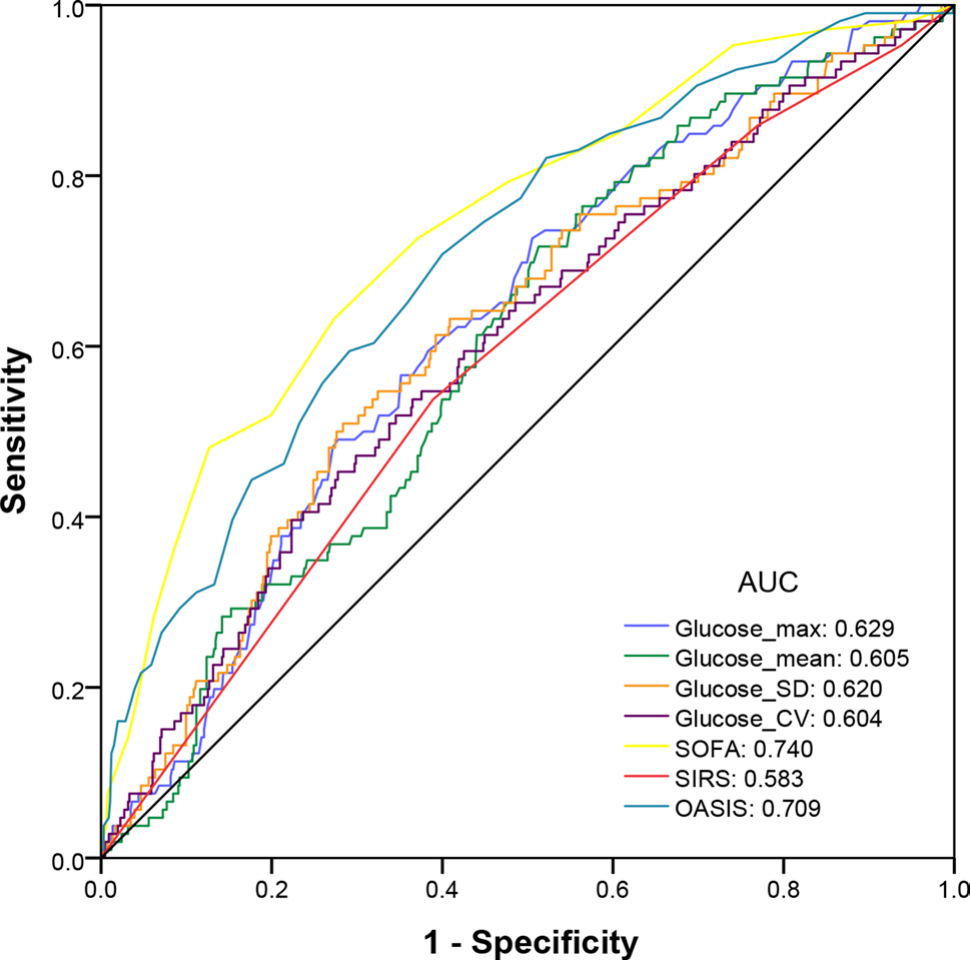
Receiver operating characteristic curves for the prediction of in-hospital mortality in patients with acute pancreatitis.

The predictive abilities of Glucose_max, Glucose_mean, Glucose_CV, and Glucose_SD were evaluated using ROC curves. According to Fig. 2, the predictive abilities of Glucose_max (AUC: 0.629), Glucose_mean (AUC: 0.605), Glucose_CV (AUC: 0.604), and Glucose_SD (AUC: 0.620) for predicting in-hospital mortality in AP patients were not as good as those of the SOFA (AUC: 0.740) and OASIS (AUC: 0.709) but better than that of the SIRS score (AUC: 0.583).

### Association between blood glucose-related indicators and in-hospital mortality

Since the coefficient of variation is obtained by dividing the standard deviation and the mean, there is a potential for collinearity. Therefore, blood glucose-related indicators were not considered as confounding variables in multiple logistic regression. The indicators showing significant differences between survivors and non-survivors (except for blood glucose-related indicators) were used as adjustment variables for the multivariate regression of Glucose_max, Glucose_mean, Glucose_CV, and Glucose_SD. In multivariate logistic regression analysis, Glucose_max, Glucose_mean, Glucose_CV, and Glucose_SD were risk factors for AP (OR 1.003, 95% CI 1.001–1.005, *P* = 0.007; OR 1.007, 95% CI 1.002–1.012, *P* = 0.011; OR 1.007, 95% CI 1.002–1.012, *P* = 0.008; OR 1.016, 95% CI 1.003–1.028, *P* = 0.013, respectively) (Table 2).

**Table 2:** Logistic regression analysis of in-hospital mortality.

	Logistic regression analysis		
	Odds ratio	95% CI	*P*-value
Glucose_max	1.003	1.001–1.004	0.008
Glucose_max**	1.003	1.001–1.005	0.007
Glucose_mean	1.006	1.001–1.010	0.014
Glucose_mean**	1.007	1.002–1.012	0.011
Glucose_SD	1.006	1.002–1.010	0.007
Glucose_SD**	1.007	1.002–1.012	0.008
Glucose_CV	1.017	1.006–1.028	0.002
Glucose_CV**	1.016	1.003–1.028	0.013

**Adjusted for age, sequential organ failure assessment score, systemic inflammatory response syndrome score, Oxford Acute Severity of Illness Score, and platelet count.

**Figure 3:**
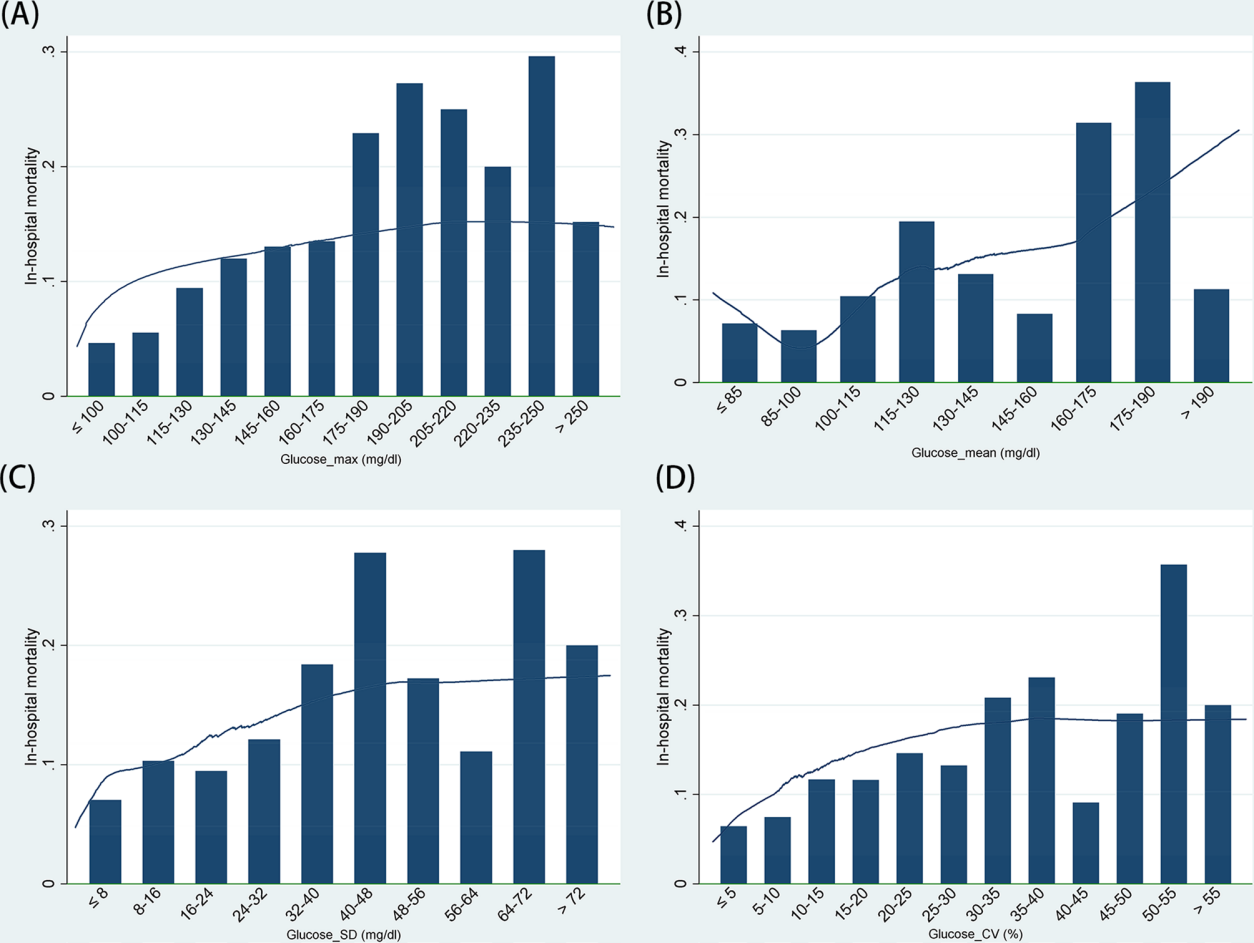
Lowess smoothing curve of the association between Glucose_max/Glucose_mean/Glucose_SD/Glucose_CV and in-hospital mortality. The Lowess smoothing curve demonstrates that there is a nonlinear relationship between (**A**) Glucose_max/(**B**) Glucose_mean/(**C**) Glucose_SD/(**D**) Glucose_CV and in-hospital mortality in patients with acute pancreatitis.

Using the Lowess smoothing curve, we found a non-linear relationship between Glucose_mean and the in-hospital mortality of AP patients. As shown in Fig. 3, the overall trend is a “U” shape. In addition, with the increase of Glucose_max, Glucose_CV, and Glucose_SD, the in-hospital mortality of AP patients showed a non-linear increase.

## Discussion

ICU patients show a critical condition, and they are in a state of physical and psychological stress. These patients are prone to stress hyperglycemia^16^ not related to diabetes. For critically ill patients with AP, there is no universally accepted insulin therapy for blood glucose control^17,18^. In this study, six indicators, namely, Glucose_initial, Glucose_max, Glucose_min, Glucose_mean, Glucose_SD, and Glucose_CV were selected as blood glucose-related indicators, and they were used to evaluate the patients’ blood glucose control levels.

Our study found that the Glucose_mean of ICU patients was correlated with mortality, producing a U-shaped curve. As shown in Fig. 3, patients with AP had the lowest in-hospital mortality when Glucose_mean was within the range of 85–100 mg/dL. This value is lower than that reported by Siegelaar et al. (7–9 mmol/L [126–162 mg/dL])^19^. This may be because Siegelaar et al. included all ICU patients, whereas we only analyzed those with AP. This probably indicates that patients with AP need to maintain their blood glucose levels within an acceptably low range rather than at the lowest possible level. However, studies have shown that hypoglycemia can increase the mortality of critically ill patients^20,21^.

Although Glucose_max reflects the patient’s acute response under stress, if the Glucose_max value is in the abnormal range, then blood glucose management is ineffective^22^. An increasing number of studies have proven that strengthening blood sugar control can reduce the in-hospital mortality risk of patients^23–25^. According to our prediction, the overall trend of the correlation between Glucose_max and in-hospital mortality should be similar to that between Glucose_mean and in-hospital mortality; however, because the number of patients who had Glucose_max levels in the hypoglycemic range in this study was extremely small, it only showed an upward trend.

There are many indicators that can be used for evaluating glucose variability, and there is no available unified standard. Hence, this study included two variability indicators: Glucose_SD and Glucose_CV. Compared to other indicators, such as glycemic lability index^26,27^ and mean amplitude of glycemic excursions^22,28^, Glucose_SD and Glucose_CV are simple, and their predictive values are similar; therefore, they are easier to use in clinical practice. In previous studies, glucose variability had a significant correlation with the mortality of ICU patients^29–31^, which concurs with our findings. In addition, we also used the Lowess smoothing curve to show the overall trend of the association between glucose variability and in-hospital mortality. Our study proves that increased blood glucose variability can lead to a poor prognosis of severe AP.

This study has some limitations. First, although our study was based on the MIMIC-III database with a large sample size, it was a single-center observational study. For reaching valid research conclusions, including many multi-center research samples is imperative. Second, owing to the characteristics of retrospective studies, we could not formulate plans for the timing and frequency of blood glucose measurement in advance. To control a consistent collection frequency, we cannot use all blood glucose values and cannot fully reflect all levels of blood glucose fluctuations. Third, for AP patients who had been admitted to the ICU on multiple occasions, only data regarding their initial admission was analyzed, which posed a potential selection bias. Hence, prospective studies with large sample sizes are warranted to provide further evidence.

In conclusion, Glucose_max, Glucose_mean, Glucose_SD, and Glucose_CV are predictive factors for in-hospital mortality in critically ill patients with AP. The results of this study need further verification through a multi-center prospective study with a larger sample size.
